# Clinical Relevance of Atrial Fibrillation in End-Stage Heart Failure Patients Actively Waiting on Heart Transplant

**DOI:** 10.3390/jcdd13050194

**Published:** 2026-04-30

**Authors:** Magda Haum, Ulrich Grabmaier, Antonia Kellnar, Christoph Müller, Korbinian Lackermair, Heidi Estner

**Affiliations:** 1 Department of Medicine I, LMU University Hospital, 81377 Munich, Germany; 2 DZHK (German Center for Cardiovascular Research), Partner Site Munich Heart Alliance, 80636 Munich, Germany; 3 Department of Cardiac Surgery, LMU University Hospital, 81377 Munich, Germany

**Keywords:** heart failure, heart transplant, atrial fibrillation, pulmonary vein ablation

## Abstract

**Background:** Recent studies have shown that catheter ablation of atrial fibrillation leads to an improvement in mortality and a reduction in hospitalization in patients with end-stage heart failure. It is therefore hypothesized that in an end-stage heart failure population, atrial fibrillation is of great relevance and that interventional therapy is crucial to preventing further progression, especially with the aim of avoiding a heart transplant. In this paper, we describe the clinical presentation of atrial fibrillation and its management in a real end-stage heart failure cohort of patients actively waiting on a heart transplant through EUROTRANSPLANT. **Methods:** A total of 577 patients have been actively listed for heart transplant in our clinic. Of these, we examined all patients who were actively listed by the key date of 31 December 2024. Patients already treated by assist devices such as the left-ventricular assist device and high-urgency listed patients were excluded, as were minors and patients in need of simultaneous transplantation of other organs in addition to the heart. **Results:** Thirty-one patients were included in our analysis. In this cohort, 18 patients (58%) had no diagnosis of atrial fibrillation or atrial flutter. A total of 13 patients (42%) presented with atrial fibrillation or flutter: 3/13 (23%) paroxysmal, 8/13 (62%) persistent, 1/13 (8%) permanent atrial fibrillation, and 1/13 (8%) atrial flutter. Moreover, 9/13 (69%) patients with atrial fibrillation had been diagnosed during evaluation and before the active listing period for heart transplant. Only three patients developed atrial fibrillation during the active listing period (two with atypical atrial flutter, one with atrial fibrillation). In those three patients, rhythm control could be achieved: the patient with new-onset atrial fibrillation was treated by pulmonary vein ablation, and in the two patients with newly diagnosed atypical atrial flutter, electrical cardioversion was performed. **Conclusions:** In our real end-stage heart failure cohort, more than half of the patients do not have atrial fibrillation. Patients diagnosed with atrial fibrillation often receive their diagnosis before they are listed for heart transplant. However, atrial fibrillation is not a common cause of clinical worsening while actively waiting on a heart transplant.

## 1. Introduction

Heart failure (HF) treatment has evolved rapidly in recent decades [1,2]. In addition to drug therapy for HF and interventional resynchronization [3,4], catheter ablation of atrial fibrillation (AF) has recently gained additional attention as an important therapeutic approach to reduce not only symptoms but also mortality [5,6]. This is particularly important given the fact that the perception of AF in end-stage heart failure has shifted significantly—from being seen merely as a symptom not worth targeting [5] to receiving growing recognition following the CASTLE and CASTLE-HTx study [6] that it may in fact represent a key therapeutic target to prevent progression into the final stage of HF.

Nevertheless, HF remains a progressive and serious disease that is associated with a limited life expectancy and increasing incidence [7]. In cases of end-stage HF, a heart transplant (HTx) is still the ultimate treatment option. Due to the shortage of donor organs in many European countries, the number of patients waiting on a HTx is high, and the waiting time for a listed patient at EUROTRANSPLANT can be many years [8,9]. Therefore, the question arises as to whether AF and its treatment are relevant early in the clinical course of end-stage HF patients or at the end of treatment during the active listing period. 

In our study, we report the baseline prevalence of AF upon admittance to the HTx waiting list and the incidence while waiting for a HTx. Furthermore, we focus on AF therapy in a real end-stage HF cohort of patients actively waiting on a HTx for end-stage HF listed by EUROTRANSPLANT.

## 2. Methods

**Figure 1:**
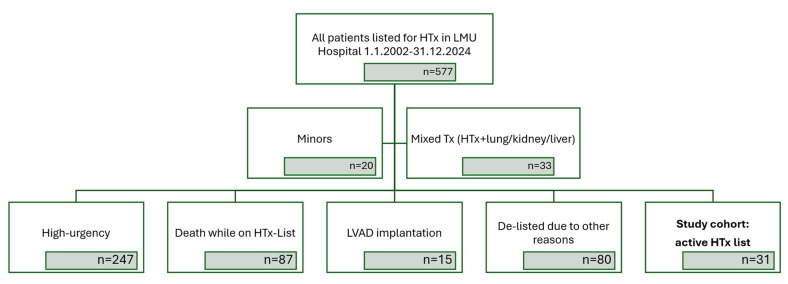
Flowchart of study screening and inclusion. All patients listed for HTx in the timespan between 1 January 2002 and 31 December 2024 were screened for study eligibility. After all exclusion criteria were met, 31 subjects were included in the analysis, as shown in bold letters. Abbreviations: HTx—heart transplant; LMU—Ludwig Maximilian University; LVAD—left-ventricular assist device; Tx—transplant.

For this study, we screened the complete cohort of all patients who had ever been listed for a HTx in our institution. Our center is the second largest HTx center in Germany, performing approximately 30 HTxs per year. We included all patients who had been placed on the EUROTRANSPLANT waiting list for a HTx in our center by the predefined key date of 31 December 2024. We excluded patients listed under high-urgency status, patients already treated by a left-ventricular assist device (LVAD), minors, and patients requiring simultaneous transplantation of other organs in addition to a HTx (Figure 1).

We assessed demographic data and clinical baseline criteria of all patients, including age, sex, underlying cardiac disease, medication, indication for HTx, and the duration of time spent on the HTx waiting list. AF diagnosis was the focus of our investigation, so time of first diagnosed AF, type of AF (paroxysmal, persistent, or permanent), and AF burden were assessed for every patient. Antiarrhythmic medication, especially whether it was primarily prescribed due to ventricular or supraventricular arrhythmia, and underlying indication were of special interest. Medical history, including procedures to achieve rhythm control, i.e., medical therapy, ablation procedures and electrical cardioversion, was documented, as well as ablation of other supraventricular arrhythmias beyond AF and ventricular arrhythmia. 

### Statistical Analysis

Statistical analysis was performed using standard descriptive methods. Continuous variables are presented as the mean ± standard deviation (SD) or median with interquartile range (IQR), as appropriate based on data distribution tested using the Shapiro–Wilk test. Categorical variables are reported as absolute numbers and percentages. No inferential statistical testing was performed, as the aim of this study was to provide a descriptive characterization of the study cohort. All analyses were conducted using SPSS software, version 31.0.2.0 (IBM Corp., Armonk, NY, USA). Data were collected prospectively over the course of regular clinical re-evaluation in the HTx outpatient clinic of our center, as requested by EUROTRANSPLANT [10], and analyzed retrospectively for this study. The dataset is complete.

## 3. Results

### 3.1. Baseline Characteristics

Thirty-one patients actively waiting on a HTx were included in our analysis. Table 1 shows the baseline characteristics of the cohort. The median age was 51 years (IQR 44–57), with 84% of patients being male and the median BMI reaching 25.7. Median time on the waiting list by 31 December 2024 was about 2.5 years (931 days, IQR 607–1527). Most patients had developed HF due to dilated cardiomyopathy (55%), followed by ischemic cardiomyopathy (29%). Less frequent reasons for a HTx were arrhythmogenic right-ventricular cardiomyopathy (ARVC) and transposition of the great arteries (TGA) (both 7% each), followed by hypertrophic cardiomyopathy (3%). 

### 3.2. Clinical Presentation and Hemodynamics

Nearly all patients (93.5%) suffered from relevant dyspnea NYHA ≥II and showed echocardiographic parameters of severe cardiac dysfunction (median left-ventricular ejection fraction of 25%, median left-ventricular end diastolic diameter of 67 mm, median TAPSE of 17.5 mm). Findings on right-heart catheterization revealed significant hemodynamic impairment with increased left-atrial pressure (median PCWP of 17 mmHg), mixed venous saturation of 65.8% and a cardiac index of 2.4. The limited hemodynamics were reflected in the laboratory findings by a pathological increase in N-terminal pro b-type natriuretic peptide (NT-proBNP 1973.5 [IQR 497.8–3504.3] pg/mL). Among valvular diseases, tricuspid and mitral insufficiency were the most common concomitant diseases. However, most patients presented only mild tricuspid and mitral insufficiency, and only five patients had undergone mitral valve treatment in the past. A total of 26 patients (87.1%) had a cardiac implantable electronic device (CIED) while waiting for a HTx, among whom 11/26 (42%) had an atrial lead implanted to allow for monitoring of atrial arrhythmia. 

### 3.3. Prevalence of Arrhythmia and Therapy

**Figure 2:**
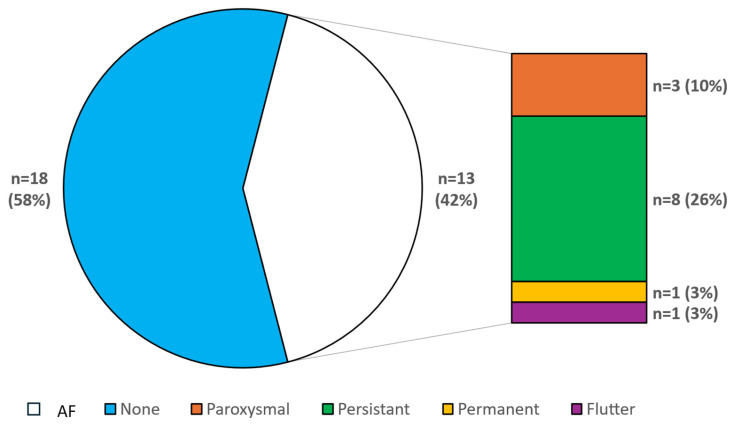
Incidence of AF in cohort of end-stage heart failure patients and distribution of atrial arrhythmias.

**Figure 3:**
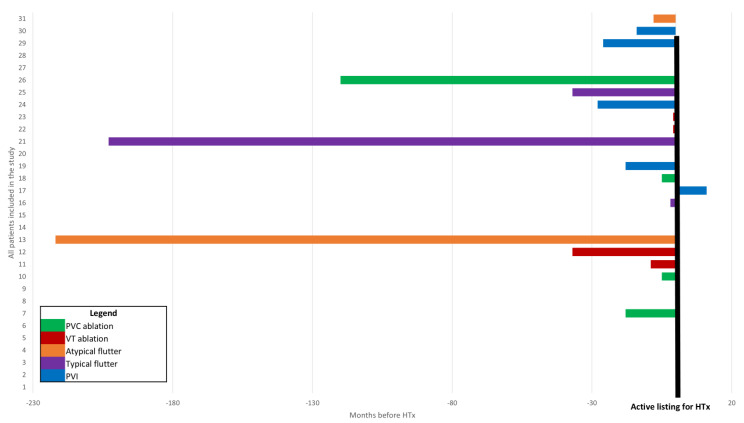
Type of ablation procedure and timing depending on the point in time at which active listing for a HTx began (marked by a black bar). All patients are consecutively listed from 1 to 31 on the y-axis. On the x-axis, timing of the procedures can be found in months. Procedure types are coded in colors, as depicted in the legend. Procedures before HTx are listed to the left and procedures after HTx are listed to the right of the black bar. If more than one ablation procedure was needed, the most recent was taken into consideration. Abbreviations: HTx—heart transplant; PVC—premature ventricular contraction; PVI—pulmonary vein ablation; VT—ventricular tachycardia.

In our cohort, 18 patients (58%) had no diagnosis of AF or atrial flutter. Of those with AF, 9 out of 13 (69%) were diagnosed before being listed for a HTx. In three patients, AF occurred paroxysmally (10%); in eight, AF was persistent (26%); one patient had permanent AF (3%); and one had atrial flutter (3%) (Figure 2). No patients with paroxysmal or persistent AF had an actual AF burden >0.1% in CIED controls. Considering the entire study cohort, antiarrhythmic pharmacological therapy consisted of betablockers (100%), amiodarone (11/31 pts. 36%), and flecainide (3%), administered in one patient due to premature ventricular contractions and amiodarone intolerance (Table 2). Nine patients were treated by amiodarone due to ventricular arrhythmias without diagnosis of AF, and two patients were treated by amiodarone only due to AF. Nine patients (29% of the cohort) were treated by ablation due to supraventricular tachycardia: four patients had ablation therapy for AF (three patients with pulmonary vein isolation and one with pulmonary vein isolation and ablation of complex fractionated atrial electrograms (CFAE)), and in five patients isthmus ablation was performed. Ablation of ventricular tachycardia was performed in six patients (19% of the cohort), and ablation of premature ventricular contraction was performed in four patients (13%). Median timing of ablation procedures was 14 months before active listing for a HTx (Figure 3). 

### 3.4. Rehospitalization

The rate of unplanned rehospitalizations in our cohort of 31 patients during the last 5 years before the key date of the study was 41.9% (13 patients). In seven patients, acute decompensation was the reason for hospital admission; ventricular arrhythmia occurred in two patients, while one suffered from acute hyperthyroidism. Rehospitalization to achieve acute rhythm control was required in two patients due to new onset of atypical atrial flutter and in one patient due to AF while on the HTx waiting list. In both cases, electrocardioversion was performed successfully, and in one patient pulmonary vein ablation was performed due to persistent AF while on the HTx list.

## 4. Discussion

This is the first study to systematically investigate the clinical impact and therapeutic options of AF in patients with end-stage HF actively waiting on a HTx.

Our investigation draws upon the results of the CASTLE-HTx study, which has been widely discussed since publication of the original data [6] and two-year follow-up data [11]. In this randomized trial, the authors reported a reduction in all-cause mortality (6% vs. 20%; HR: 0.29; *p* < 0.05), LVAD implantation (1% vs. 10%; *p* < 0.05), and highly urgent HTx (1% vs. 6%; *p* > 0.05) and an improvement in left-ventricular ejection fraction after 12 months (+8% vs. +1%; *p* < 0.05) due to a reduction in AF burden (−31% vs. −9%; *p* < 0.05) as a result of AF ablation. One of the inclusion criteria specified referral to a HTx center for evaluation of HTx. At first sight, this trial suggests that effective interventional treatment of AF might be the key factor in patients with end-stage heart failure to prevent LVAD implantation and transplantation. In this monocentric study performed in the largest heart transplant center in Germany, with approximately 80 HTxs per year, patients referred for HTx evaluation were screened for study participation. However, it was unclear how many patients not only presented with but also were actually listed for HTx in this monocentric cohort. 

In our study, we present the incidence and clinical impact of diagnosed AF in a real end-stage HF cohort defined by actively waiting on a HTx for end-stage HF as listed by EUROTRANSPLANT in the second largest HTx center in Germany. It is important to emphasize that in our cohort, patients had already developed end-stage HF after exhausting all therapeutical options, including medication, device therapy and rhythm control. 

In our large transplant center in Germany, which performed 27 HTxs in 2022, 31 adult patients were awaiting a HTx on non-highly urgent basis at the time of the study. At the time of joining the waiting list for a HTx, 18 patients (58%) had not been diagnosed with AF. One patient had been diagnosed with permanent AF. In the other 12 patients, AF was persisting or paroxysmal without actual AF burden, as defined by an AF burden >1%. The beneficial effects of interventional treatment of symptomatic AF in stable HF patients have been elucidated by the CASTLE AF trial [5].

Our data show an overall low AF burden while waiting for a HTx and successful rhythm control therapy in most patients in advance. These findings are consistent and clinically “in line” with data shown in CASTLE-HTx, where a relevant rate of successful AF therapy was achieved, although, of course, no causal inferences regarding treatment effects can be drawn from our descriptive study. However, the clinical observations from our cohort are consistent with the results of the randomized CASTLE-HTx study and therefore support the study’s key finding that effective interventional treatment of symptomatic AF should be part of standard treatment among HF patients and should not be left to progress to “end stage”. Our data indicate that AF is not a frequent factor in progression of HF to end-stage HF requiring a HTx, as AF often manifests at a considerably earlier stage. Furthermore, the novelty of our finding is that not all patients develop AF, even those with terminal HF.

In this study, patients were included based on a set date. This was based on the assessment that the treatment of AF had only recently become a main focus, and we wanted to include a group of transplant patients who had received the most modern treatment available. Therefore, we only included a relatively small subgroup of all HTx patients on the waiting list. Patients with LVADs were deliberately excluded from the present cohort describing AF management. This patient population represents a distinct clinical entity due to altered hemodynamics under continuous-flow support and the need for specific, mandatory anticoagulation strategies. Furthermore, the pathophysiology of AF and the therapeutic objectives in the LVAD setting differ substantially from those in the general population, particularly regarding rhythm versus rate control and the role of atrial contraction. Interventional approaches such as catheter ablation are also associated with unique technical challenges and potentially increased risks in this group. Including LVAD patients would therefore have reduced the homogeneity of the cohort and limited the interpretability of the results with respect to standardized AF management strategies in broader clinical practice.

With this study, data on AF in a cohort actively waiting on a HTx are provided for the first time. Yet limitations must be considered when interpreting the results of this observational study. Most important, the analysis is retrospective and includes data from a single center. Our cohort shows a relatively low prevalence of AF, which can limit the interpretability of the results, but it should be interpreted in the context of prior treatment, as many patients had already received therapy for AF earlier in the course of their disease. As a result of careful patient selection, excluding patients with LVADs due to different considerations regarding invasive procedures such as ablation, patients requiring transplantation of other organs alongside a HTx, minors, and patients waiting for a high-urgency HTx, the small patient group is not suitable for yielding generalizable results. However, for this highly specialized patient subgroup with terminal HF, data on the clinical impact of AF are missing and are urgently needed, as waiting time for a HTx remains long in many European countries. 

## 5. Conclusions

In a real end-stage HF cohort, AF is often diagnosed in the course of disease progression, and nearly half of the patients have already been diagnosed with AF by the time they’re listed for a HTx. Ablation strategies and pharmaceutical options are important in the treatment of HF to postpone deterioration and are performed in clinical practice much earlier than the onset of end-stage HF. However, AF is not a common cause of clinical worsening or rehospitalization while actively waiting on a HTx.

## 6. Clinical Perspectives

What is known? 

Recent studies have shown that catheter ablation of AF leads to an improvement in mortality and a reduction in hospitalization in patients with end-stage HF. It is therefore hypothesized that in an end-stage HF population, AF is of great relevance and that interventional therapy is crucial in preventing further progression, especially with the aim of avoiding a HTx. 

What is new? 

AF is often diagnosed in patients with HF, and nearly half of the patients on the HTx waiting list have received an AF diagnosis. However, it is not a common cause of clinical worsening or rehospitalization while waiting for a HTx.

What is next? 

In clinical practice, ablation strategies and optimized drug therapy should be implemented early in HF management to stabilize patients and delay progression to end-stage disease. Patients on the HTx waiting list should be screened for AF regularly with the aim of achieving rhythm control.

## Figures and Tables

**Table 1:** Baseline characteristics of patients actively waiting on a HTx.

Baseline Characteristics	Total Population (*n* = 31)
Age (in years)	51 (IQR 44–57)
Male gender	83.9% (26)
BMI	25.7 (IQR 23.0–28.6)
Time since HF diagnosis until HTx list (in years)	8 (IQR 3–14)
Time on HTx waiting list by 31 December 2024 (in days)	931 (IQR 607–1527)
Reason for HF	
Ischemic cardiomyopathy	29.0% (9)
Dilated cardiomyopathy	54.8% (17)
Hypertrophic cardiomyopathy	3.2% (1)
Arrhythmogenic right-ventricular cardiomyopathy	6.5% (2)
Transposition of the great arteries	6.5% (2)
NYHA	
<III	35.5% (11)
≥III	64.5% (20)
Echocardiographic Parameters	
LVEF (in %)	25.0 (IQR 20.0–27.0)
LVEDD (in mm)	67.0 (IQR 61.75–74.0)
TAPSE (in mm)	17.5 (IQR 13.0–22.0)
Hemodynamic Parameters According to Right-Heart Catheterization	
PCWP (mean)	17 (IQR 10–22)
Mixed venous saturation (in %)	65.8 (IQR 61.0–68.5)
Cardiac Index	2.4 (IQR 1.75–2.6)
Cardiac Implantable Electronic Devices	
VVI-ICD	38.7% (12)
VDI-ICD	3.2% (1)
DDD-ICD	9.7% (3)
Subcutaneous ICD	9.7% (3)
CRT defibrillator	22.6% (7)
CRT pacer	3.2% (1)
No cardiac implantable electronic device	12.9 % (4)
Laboratory Findings	
Hemoglobin (g/dL)	14.4 (IQR 13.2–15.4)
Creatinine (mg/dL)	1.2 (IQR 1.1–1.4)
Glomerular filtration rate (ml/min)	66.0 (IQR 47.0–77.5)
Lactate (mmol/L)	1.5 (IQR 1.3–2.0)
Bilirubin (mg/dL)	0.8 (IQR 0.5–1.2)
Aspartate aminotransferase (U/L)	26.0 (IQR 21.3–34.3
Alanine aminotransferase (U/L)	26.0 (IQR 22.0–34.0)
proBNP (pg/ml)	1973.5 (IQR 497.8–3504.3)
FEV1 (in %)	3.01 (IQR 2.65–3.64)
Comorbidities	
Chronic kidney disease grade >G2	41.9% (13)
Dialysis	3.2% (1)
Aortic stenosis	0
Aortic insufficiency	12.9% (4)
* None	87.1% (27)
* Mild	9.7% (3)
* Moderate	3.2% (1)
* Severe	0
Mitral stenosis	0
Mitral insufficiency	64.5% (20)
* None	35.5% (11)
* Mild	41.9% (13)
* Moderate	9.7% (3)
* Severe	12.9% (4)
Tricuspid insufficiency	90.3% (28)
* None	9.7% (3)
* Mild	41.9% (13)
* Moderate	29.0% (9)
* Severe	19.4% (6)
History of aortic valve treatment	0
History of mitral valve treatment	16.1% (5)
History of tricuspid valve treatment	0
Diabetes mellitus	16.1% (5)

BMI—body mass index; CRT—cardiac resynchronization therapy; DDD—dual-chamber antibradycardia (pacer/defibrillator); FEV1—forced expiratory volume in 1 second; GFR—glomerular filtration rate; HF—heart failure; HTx—heart transplantation; ICD—implantable cardioverter–defibrillator; IQR—interquartile range; LVEDD—left-ventricular end-diastolic diameter; LVEF—left-ventricular ejection fraction; NYHA—New York Heart Association; PCWP—pulmonary capillary wedge pressure; proBNP—probrain natriuretic peptide; TAPSE—tricuspid annular plane systolic excursion; VDI—single-chamber antibradycardia (pacer/defibrillator) with atrial sensing channel; VVI—single-chamber antibradycardia (pacer/defibrillator).

**Table 2:** Therapeutic measures for treatment of arrhythmia.

Antiarrhythmic Therapy	Total Population (*n* = 31)
Antiarrhythmic Pharmacological Therapy	
Amiodarone	35.5% (11)
* Indication for therapy of VT/PVC	29.0% (9)
* Indication for therapy of AF	6.5% (2)
Betablockers	100.0% (31)
Flecainide	3.2% (1)
History of Ablation to Treat	
Supraventricular tachycardia	29.0% (9)
* AF ablation	12.9% (4)
>Pulmonary vein isolation	9.7% (3)
>Pulmonary vein isolation and substrate modification	3.2% (1)
* Isthmus ablation	16.1% (5)
Ventricular tachycardia	19.4% (6)
Premature ventricular contraction	12.9% (4)

## Data Availability

The data underlying this article will be shared upon reasonable request to the corresponding author.

## Abbreviations

AF	Atrial fibrillation
CIED	Cardiac implantable electronic device
CFAE	Complex fractionated atrial electrogram
DCM	Dilative cardiomyopathy
HCM	Hypertrophic cardiomyopathy
HF	Heart failure
HTx	Heart transplantation
LVAD	Left-ventricular assist device
NYHA	New York Heart Association
NT-proBNP	N-terminal pro b-type natriuretic peptide

## References

[1] McDonagh T.A., Metra M., Adamo M., Gardner R.S., Baumbach A., Böhm M., Burri H., Butler J., Čelutkienė J., Chioncel O. (2021). 2021 ESC Guidelines for the diagnosis and treatment of acute and chronic heart failure. Eur. Heart J..

[2] Newman J.D., O’mEara E., Böhm M., Savarese G., Kelly P.R., Vardeny O., Allen L.A., Lancellotti P., Gottlieb S.S., Samad Z. (2024). Implications of Atrial Fibrillation for Guideline-Directed Therapy in Patients with Heart Failure. JACC.

[3] Karlström P., Pivodic A., Dahlström U., Fu M. (2024). Modern heart failure treatment is superior to conventional treatment across the left ventricular ejection spectrum: Real-life data from the Swedish Heart Failure Registry 2013–2020. Clin. Res. Cardiol..

[4] Moysidis D.V., Kartas A.M., Samaras A., Papazoglou A.S., Patsiou V., Bekiaridou A., Baroutidou A., Tsagkaris C., Karagiannidis E., Daios S. (2023). Prescription Rates and Prognostic Implications of Optimally Targeted Guideline-Directed Medical Treatment in Heart Failure and Atrial Fibrillation: Insights From The MISOAC-AF Trial. J. Cardiovasc. Pharmacol..

[5] Marrouche N.F., Kheirkhahan M., Brachmann J. (2018). Catheter Ablation for Atrial Fibrillation with Heart Failure. N. Engl. J. Med..

[6] Sohns C., Fox H., Marrouche N.F., Crijns H.J., Costard-Jaeckle A., Bergau L., Hindricks G., Dagres N., Sossalla S., Schramm R. (2023). Catheter Ablation in End-Stage Heart Failure with Atrial Fibrillation. N. Engl. J. Med..

[7] Khan M.S., Shahid I., Bennis A., Rakisheva A., Metra M., Butler J. (2024). Global epidemiology of heart failure. Nat. Rev. Cardiol..

[8] Annual Report of Eurotransplant. https://www.eurotransplant.org/statistics/annual-report/.

[9] Critsinelis A., Karamchandani M.M., Hironaka C.E., Nordan T., Chen F.Y., Couper G.S., Kawabori M. (2023). Heart Transplant Waitlist Outcomes and Wait Time by Center Volume in the Pre-2018 Allocation Change Era. Asaio J..

[10] (2016). Eurotransplant Manual. https://www.eurotransplant.org/allocation/eurotransplant-manual.

[11] Sohns C., Moersdorf M., Marrouche N.F., Bergau L., Costard-Jaeckle A., Crijns H.J., Fox H., Hindricks G., Dagres N., Sossalla S. (2024). Catheter Ablation in Patients With End-Stage Heart Failure and Atrial Fibrillation: Two-Year Follow-Up of the CASTLE-HTx Trial. Circulation.

